# Platelet satellitism in chronic renal failure: an experimental study

**Published:** 2015-01-01

**Authors:** Vibha Gupta, Shahbaj Ahmad, Harish Chandra, Vikas Shrivastava

**Affiliations:** ^1^Department of Pathology, Himalayan Institute Medical Sciences, Swami Ram Nagar, Dehradun, India; ^2^Department Nephrology, Himalayan Institute Medical Sciences, Swami Ram Nagar, Dehradun, India

**Keywords:** Platelet satellitism, Heparin, Chronic kidney disease, Platelet rosettes, Dialysis, Diabetes mellitus

## Abstract

The phenomenon of platelet satellitism (PS) is characterized by formation of platelet rosettes around polymorphonuclear leukocytes. We present a case of chronic Kidney disease that developed PS after second cycle of dialysis. A group of experiments have been conducted that suggested that contrary to common belief, anticoagulant does not play any role. In view of available literature we suggest that this phenomenon could be related to either or both of the hemodynamic stress and biocompatibility of the dialysis membrane.

**Implication for health policy/practice/research/medical education:**

The phenomenon of platelet satellitism (PS) is characterized by formation of platelet rosettes around polymorph- nuclear leukocytes. We suggest that this phenomenon could be related to either or both of the hemodynamic stress and biocompatibility of the dialysis membrane.

## Introduction

The rare phenomenon of platelet satellitism (PS) (less than 100 cases since 1963 characterized by formation of platelet rosettes around polymorphonuclear (PMN) leukocytes was first described as *in vitro* phenomenon in 1963 (1). PS have been reported, not only among healthy but also in many diseases such as cutaneous vasculitis, mantle cell lymphoma, chronic lymphoid leukaemia, urinary tract infection (2-7). However, no specific relationship could be established between PS and the medical conditions, except its association with ethylene–di-amine–tetra-acetic-acid (EDTA) anticoagulant (8-10).

We present a case of PS in chronic kidney disease (CKD) stage-5 on dialysis, still a non-reported medical condition. More ever, an attempt is made through various observation and experiments to relate already existing concept of ‘stress Platelets’ seen with PS to bio-incompatibility of dialysis membrane, and its occurrence in heparinized blood instead of EDTA. PS is associated with false thrombocytopenia and may result in inadvertent splenectomy or transfusion with platelet concentrates (11,12).

## Case presentation

A 60-year-old male with diabetes and CKD underwent first haemodialysis. During initial two visits, blood urea nitrogen and creatinine were raised with low hemoglobin of 7.6 g/dl and normal platelet counts (Table 1).

**Table 1:** Biochemical and haematological parameters at first two visits

**Parameters**	**Pre-dialysis**	**Post-dialysis**
**Visit 1**		
BUN (mg/dl)	44	18
Creatinine (mg/dl)	8.26	3.27
WBC/μL	7070	7100
Platelets/μL	166	180
**Visit 2**		
BUN (mg/dl)	35	12
Creatinine (mg/ dl)	8.39	3.50
WBC/μL	6011	6180
Platelets/μL	160	170

Before third dialysis, the analyzer gave flag for low platelets. Leishman’s stained slides showed a garland arrangement of platelets in number of two to six around most of the neutrophils while other areas showed platelets aggregates (Figure 1.1).

**Figure 1:**
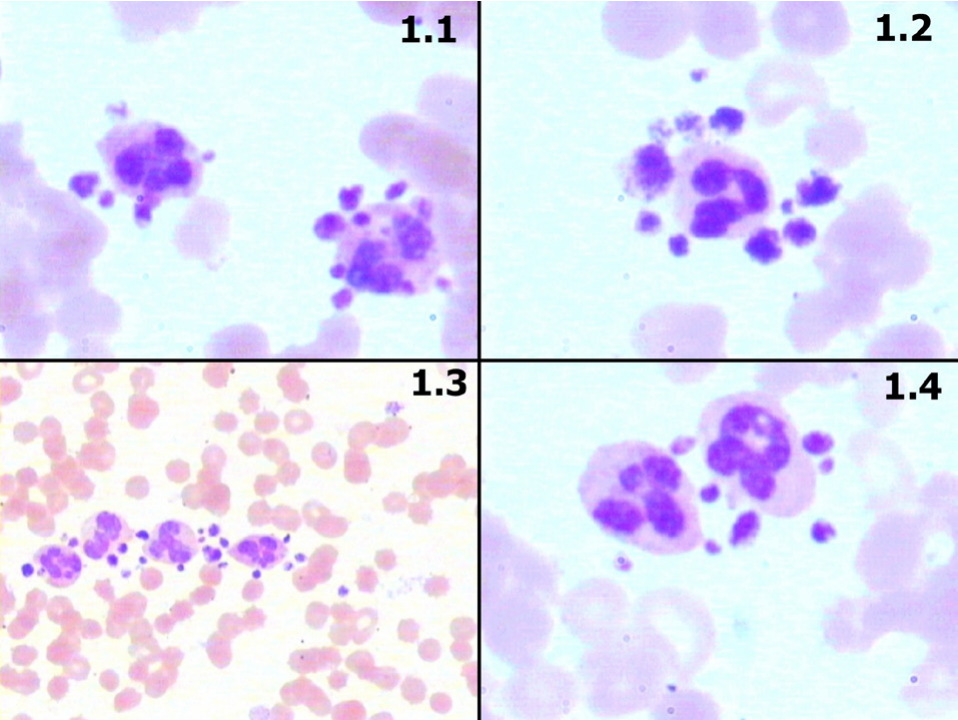
**Platelet satellitism in the peripheral smears(Leishman stained)**. 1.1 Demonstrate PS in blood collected in EDTA anticoagulant under 100X (oil immersion). 1.2 Demonstrate PS in blood collected in Heparin anticoagulant 100 X (oil immersion). 1.3 Demonstrate PS in blood collected in EDTA anticoagulant under 10X. 1.4 Demonstrate PS in direct finger prick blood smear prepared 100 X (oil immersion)

Peripheral blood slides of initial two visits were then reviewed which did not show PS. It was concluded that it was at the third visit when the PS was observed for the first time. The reason for the PS was investigated and experiments were conducted.

### Investigations and experiments

We found that between first and third visit, he was transfused with one unit of packed red blood cell (PRBC). There was a possibility that antibodies could have been transmitted along with PRBC which had induced PS (4). To test this possibility, we mixed the corresponding plasma of the blood unit, from which the PRBC was prepared in this patient, with the leukocyte rich plasma of a healthy donor of the matched blood group. Leishman’s stained slides were prepared which failed to demonstrate the phenomenon of PS. After this, the plasma of this patient (taken in EDTA vial) was also incubated with the matched group leukocyte rich plasma and slides were prepared, which also failed to demonstrate PS.

Further experiments were conducted to identify the role of anticoagulants during subsequent visits. Blood samples were taken in EDTA, heparin vacutainers (BD) and direct smears were also prepared as control. Confounding factors like altered ratio of anticoagulant and blood were taken care of which could induce clumping of platelets. Hematological parameters were comparable between EDTA and heparin anticoagulants (hemoglobin 9.1 and 9.0 gm/dl. Total leukocyte count; 5182/µL and 3874/µL. Total platelets; 68,000/µL and 74880/µL, respectively). Observations in peripheral blood smears are depicted Figure 1.

## Discussion

This case showed that PS developed after second cycle of dialysis in a CKD patient. PRBC transfusion was not responsible for PS. PS was not only seen in blood collected in EDTA vial but also when heparin was used an anticoagulant. Lastly, PS was seen not only *in vitro* but also in direct smears. Thus, the PS could be pathophysiologically related either to CKD or dialysis but unrelated to EDTA.

### Role of anticoagulant

Literature is available in favour of the notion that PS is induced only by EDTA anticoagulant by exposing the antigen on the surface of both platelet and neutrophils (8-10). However, contradictory literature is also available demonstrating PS even with heparin anticoagulant (4) and *in vivo* (13). Almquist *et al*. (13) took blood sample in siliconized vacutainers tubes containing 3.8% sodium citrate (BD) instead of EDTA vials and demonstrated that PS was found *in vivo* in cases of CKD with DM. This was not seen in subjects with DM with normal kidney function (13). The results of our experiments also support the observations that PS is not only seen with anticoagulant EDTA, but also with heparin, and *in vivo*. These observations raised the question whether PS was solely due to the stimulant effect of EDTA (8-10). More studies are required to investigate it.

### Role of chronic kidney disease

Studies have shown that patients of CKD with DM are at higher risk of atherosclerosis and hemodynamic stress due to increased inflammatory activity. Almquist *et al*. (13) have evidenced the same through flow cytometry, which could produce “stress platelets”. Payne (7) showed that platelets participating in PS have ultra-structural changes which were similar to that seen after giving one dose of to healthy people. Increased number of glycogen particles was seen in the cytoplasm of platelets in both these conditions and these were labelled as ‘stress platelets’. He proposed that cases with PS had history of either bleeding or thrombosis, a hemodynamic stress which could have resulted in consumption of platelets and production of ‘stress platelets’ (7). We hypothesized that present patient was also under hemodynamic stress that resulted in PS, *in vivo*. Perhaps, when the dialysis was started in this patient, might be the ‘stress platelets’ were already circulating in his blood.

### Role of dialysis

If stress platelets were already in circulation, why the PS appeared only after two haemodialysis? Literature suggests that bio-incompatibility of dialysis membrane also plays an important role. During dialysis when blood comes in contact with membrane, leukocytes, platelets and complement system might be activated leading to platelet adhesion, aggregation and coagulation (14,15). We used cellulose-acetate dialysis membrane which is known to have greater bio-incompatibility, as compared with synthetic membrane e.g. polysulfone (14). We hypothesized that in our case, already circulating stress-platelets, which were present even before dialysis, reached a critical number after two times exposure to a cellulose-acetate dialysis membrane and precipitated the phenomenon of PS.

### Packed red blood cell transfusion was not responsible to transmit the phenomenon

We could not demonstrate that the phenomenon of PS in our case was acquired through PRBC. Contradictory observations are also available which reported that PS could be induced in the recipient when the blood of the patient having PS is transfused (4,16). Hence, more studies are required to investigate role of packed red blood cells along with other blood components in transmission of phenomenon of PS.

## Conclusion

CKD with associated DM can induce hemodynamic changes, causing the generation of “stress platelets” which may be responsible for PS. Bio-incompatibility of the dialysis membrane further stimulates this process, resulting in precipitation of platelet satellitism. We also suggest that platelet aggregates should be checked in the peripheral blood during the earlier cycles of dialysis, so that membrane incompatibility can be taken care of. However further studies are required, taking into consideration the duration of illness, relationship with diabetes and other parameters.

## Authors’ contributions

VG and SA were involved in the conception and designing of the study. VG, HC and VS acquired and analyzed the data. VG drafted the manuscript and other authors provided the critical scientific content. Final draft was approved by all authors.

## Conflict of interests

The authors declared no competing interests.

## Ethical considerations

Ethical issues (including plagiarism, misconduct, data fabrication, falsification, double publication or submission, redundancy) have been completely observed by the authors.

## Funding/Support

None.
